# Circulating Inflammatory Markers and Mammographic Density Phenotypes: a Mendelian Randomization Analysis

**DOI:** 10.64898/2026.09.08.26362541

**Published:** 2026-09-10

**Authors:** Austin Hammermeister Suger, Tabitha A. Harrison, Cameron B. Haas, Hongjie Chen, Shaoqi Fan, Manjeet K. Bolla, Qin Wang, Joe Dennis, Kyriaki Michailidou, Alison M. Dunning, Douglas F. Easton, Melissa C. Southey, Peter A. Fasching, Lothar Haeberle, Jennifer Stone, Manuela Gago-Dominguez, Jose E. Castelao, Kristan Aronson, Rachel A. Murphy, D. Gareth Evans, Sherene Loi, kConFab Investigators, Fergus J. Couch, Siddhartha Yadav, Roger L. Milne, Christopher A. Haiman, Christopher G. Scott, Aaron Norman, Esther M. John, Anna Marie Mulligan, Vessela N. Kristensen, A. Heather Eliassen, Irene L. Andrulis, Jonine D. Figueroa, Susan Astley, Kamila Czene, Paul D.P. Pharoah, Antonis C. Antoniou, Montserrat García-Closas, Amy Berrington, Gretchen L. Gierach, Celine M. Vachon, Rulla M. Tamimi, Sara Lindström

**Affiliations:** 1 Department of Epidemiology, University of Washington School of Public Health, Seattle, WA, USA; 2 Division of Cancer Epidemiology and Genetics, National Cancer Institute, Bethesda, MA, USA; 3 Centre for Cancer Genetic Epidemiology, Department of Public Health and Primary Care, University of Cambridge, Cambridge, UK; 4 Biostatistics Unit, The Cyprus Institute of Neurology and Genetics, Nicosia, Cyprus; 5 Centre for Cancer Genetic Epidemiology, Department of Oncology, University of Cambridge, Cambridge, UK; 6 Precision Medicine, School of Clinical Sciences at Monash Health, Monash University, Clayton, Victoria, Australia; 7 Department of Clinical Pathology, The University of Melbourne, Melbourne, Victoria, Australia; 8 Department of Medicine, St Vincent’s Hospital, The University of Melbourne, Fitzroy, Victoria, Australia; 9 Department of Gynecology and Obstetrics, Comprehensive Cancer Center Erlangen-EMN, Friedrich-Alexander University Erlangen-Nuremberg, University Hospital Erlangen, Erlangen, Germany; 10 Genetic Epidemiology Group, School of Population and Global Health, University of Western Australia, Perth, Western Australia, Australia; 11 Cancer Genetics and Epidemiology Group, Genomic Medicine Group, Instituto de Investigación Sanitaria de Santiago de Compostela (IDIS), Complejo Hospitalario Universitario de Santiago, SERGAS, Santiago de Compostela, Spain; 12 Oncology and Genetics Unit, Instituto de Investigación Sanitaria Galicia Sur, SERGAS, Vigo, Spain; 13 Public Health Sciences, Queen’s University, Kingston, ON, Canada; 14 School of Population and Public Health, University of British Columbia, Vancouver, BC, Canada; 15 Population Health Sciences, BC Cancer, Vancouver, BC, Canada; 16 Division of Evolution, Infection and Genomic Sciences, School of Biological Sciences, Faculty of Biology, Medicine and Health, University of Manchester, Manchester Academic Health Science Centre, Manchester, UK; 17 North West Genomics Laboratory Hub, Manchester Centre for Genomic Medicine, St Mary’s Hospital, Manchester University NHS Foundation Trust, Manchester Academic Health Science Centre, Manchester, UK; 18 Division of Cancer Research, Peter MacCallum Cancer Centre, Melbourne, Australia; 19 The Sir Peter MacCallum Department of Medical Oncology, The University of Melbourne, Parkville, Australia; 20 Department of Laboratory Medicine and Pathology, Mayo Clinic, Rochester, MN, USA; 21 Department of Medical Oncology, Mayo Clinic, Rochester, MN, USA; 22 Cancer Epidemiology Division, Cancer Council Victoria, Melbourne, Victoria, Australia; 23 Centre for Epidemiology and Biostatistics, Melbourne School of Population and Global Health, The University of Melbourne, Melbourne, Victoria, Australia; 24 Department of Preventive Medicine, Keck School of Medicine, University of Southern California, Los Angeles, CA, USA; 25 Department of Quantitative Health Sciences, Division of Clinical Trials and Biostatistics, Mayo Clinic, Rochester, MN, USA; 26 Department of Quantitative Health Sciences, Division of Epidemiology, Mayo Clinic, Rochester, MN, USA; 27 Departments of Epidemiology & Population Health and of Medicine (Oncology), Stanford University School of Medicine, Stanford, CA, USA; 28 Stanford Cancer Institute, Stanford University School of Medicine, Stanford, CA, USA; 29 Department of Laboratory Medicine and Pathobiology, University of Toronto, Toronto, Ontario, Canada; 30 Laboratory Medicine Program, University Health Network, Toronto, Ontario, Canada; 31 University of Connecticut, Storrs, CT, USA; 32 Department of Cancer Genetics, Institute for Cancer Research, Oslo University Hospital-Radiumhospitalet, Oslo, Norway; 33 Channing Division of Network Medicine, Department of Medicine, Brigham and Women’s Hospital and Harvard Medical School, Boston, MA, USA; 34 Departments of Nutrition and Epidemiology, Harvard T.H. Chan School of Public Health, Boston, MA, USA; 35 Department of Epidemiology, Harvard T.H. Chan School of Public Health, Boston, MA, USA; 36 Fred A. Litwin Center for Cancer Genetics, Lunenfeld-Tanenbaum Research Institute of Mount Sinai Hospital, Toronto, Ontario, Canada; 37 Department of Molecular Genetics, University of Toronto, Toronto, Ontario, Canada; 38 Usher Institute of Population Health Sciences and Informatics, The University of Edinburgh, Edinburgh, UK; 39 Cancer Research UK Edinburgh Centre, The University of Edinburgh, Edinburgh, UK; 40 Division of Cancer Epidemiology and Genetics, National Cancer Institute, National Institutes of Health, Department of Health and Human Services, Bethesda, MD, USA; 41 Division of Informatics, Imaging and Data Science, University of Manchester, Manchester, UK; 42 Department of Medical Epidemiology and Biostatistics, Karolinska Institutet, Stockholm, Sweden; 43 Department of Computational Biomedicine, Cedars-Sinai Medical Center, West Hollywood, CA, USA; 44 Department of Public Health and Primary Care, University of Cambridge, Cambridge, UK; 45 Division of Genetics and Epidemiology, The Institute of Cancer Research (ICR), London, UK; 46 Cancer Epidemiology and Prevention Research Unit, The Institute of Cancer Research (ICR) and Imperial College London, London, UK; 47 Department of Population Health Sciences, Weill Cornell Medicine, New York, NY, USA; 48 Public Health Sciences Division, Fred Hutchinson Cancer Research Center, Seattle, WA, USA

## Abstract

Observational studies have identified associations between circulating inflammatory markers (CIMs) and mammographic density (MD) phenotypes, but whether these associations reflect causal effects remains unclear. We utilized two-sample Mendelian randomization (MR) to assess relationships between 60 CIMs and MD phenotypes in individuals of European genetic ancestry by examining associations between genetically predicted CIM concentrations and three MD phenotypes (dense area, DA: *N* = 13,965; non-dense area, NDA: *N* = 14,036; percent density, PMD: *N* = 17,841). Primary analyses were conducted using the inverse variance weighted MR method. No CIM-MD phenotype associations reached statistical significance after correction for multiple testing. At a nominal significance level of *p* < 0.05, we observed suggestive evidence for eight associations represented by six unique CIMs (MD phenotype/direction of association): CCL23 (DA/+), CD40 (NDA/−), GDNF (DA/+ & PMD/+), MMP-10 (DA/+ & PMD/+), TRANCE (DA/−), and TWEAK (NDA/+). In multivariable MR analyses accounting for body mass index (BMI), we observed six nominal associations represented by six unique CIMs, although most instruments demonstrated weak conditional instrument strength after inclusion of BMI. Overall, our comprehensive analysis of 60 CIMs provides limited evidence supporting broad causal effects of circulating inflammatory markers contributing to variation in MD phenotypes.

## Introduction

Female breast cancer is the most diagnosed cancer among women worldwide, with an estimated 2.3 million incident cases diagnosed in 2022, and remains a major contributor to cancer mortality [1]. Mammographic density (MD), defined by the relative amounts of connective, epithelial, and adipose tissue in the breast, is a well-established risk factor for breast cancer [2–4]. MD phenotypes include mammographic dense area (DA), non-dense area (NDA), and percentage of dense area in the breast (percent mammographic density; PMD). In addition to being a breast cancer risk factor, increased MD (higher PMD or DA) is associated with reduced sensitivity and specificity for mammography screening [5,6]. Characterizing factors associated with MD could help us better understand its influence on breast cancer risk and impact on screening.

Tumor-promoting inflammation is a cancer hallmark [7,8] and circulating inflammatory markers (CIMs) have been associated with breast cancer in molecular and observational epidemiological studies [9–11]. One of the most studied CIMs is C-reactive protein (CRP), which has a functional role in inflammatory processes [12], with studies supporting its involvement in apoptosis, immune cell recruitment, and lipid accumulation [13]. Multiple studies have observed an association between higher concentrations of circulating CRP and risk of breast cancer [14–17], yet many of the identified associations were modest and varied by body mass index (BMI), menopausal status, or other factors. Relationships between other CIMs and breast cancer risk have been studied less extensively and the associations that have been detected are often inconsistent across studies and vary by breast cancer subtype, BMI, menopausal status, menopausal hormone therapy use, or other factors [18–20].

Elucidating the relationships between CIMs and breast cancer risk factors could help disentangle the etiological role of systemic inflammation in breast cancer risk. Studies have identified associations between MD phenotypes and circulating concentrations of interleukin 6 (IL-6) [21–23], interleukin 8 (IL-8) [22,23], tumor necrosis factor receptor 2 (TNFR2) [24], vascular endothelial growth factor (VEGF) [23], and CRP [21,22,24,25]. However, similar to the breast cancer studies, many associations were inconsistent across studies and varied by potential confounding factors [21,25].

The objective of this study was to comprehensively assess associations between genetically predicted CIMs and MD phenotypes using a two-sample Mendelian randomization (MR) approach, as MR can help mitigate some methodological limitations of observational studies, such as reverse causation and uncontrolled confounding. We further performed multivariable MR analyses including genetically-predicted BMI as an exposure, given its strong association with MD phenotypes and involvement in inflammatory processes [4,26]. Previous bidirectional MR studies of CRP and BMI have suggested that BMI is likely to affect CRP, rather than vice versa, and thus BMI is a potential confounder in CRP-MD phenotype relationships [27,28]. In total, we conducted MR analyses of 60 genetically predicted CIM exposures in relation to DA, NDA, and PMD phenotypes.

## Methods

### MR study design and data sources

The STROBE-MR checklist was used to guide the reporting of our MR analyses [29]. Causal diagrams illustrating the relationships that we investigated using MR and multivariable MR analyses are shown in Figure 1. We used summary statistics from published European genetic ancestry GWAS for BMI (*N subjects* = 461,460) [30], circulating CRP (*N* = 575,531) [31] and TNFR2 (*N* = 21,758) [32], and circulating levels of 58 additional inflammation-related proteins (*N* = 14,824) for which at least one genome-wide significant (*p* < 5×10^−8^) single nucleotide polymorphism (SNP) exists [33] (Table S1), for a total of 60 unique CIMs. GWAS summary statistics were obtained from either the IEU OpenGWAS [34,35], the EBI-NHGRI GWAS Catalog [36], or studyspecific repositories [33], selecting the largest available European genetic ancestry GWAS when multiple were available. For MD phenotypes (DA: *N* = 13,965; NDA: *N* = 14,036; PMD: *N* = 17,841), we used GWAS summary statistics from the Breast Cancer Association Consortium (BCAC) dataset described in Haas et al., 2024 [37] (Table S2). The original GWAS or studies meta-analyzed by those GWAS obtained ethical approval from their organization/institution(s) ethical review boards as described in the cited articles. The GWAS summary statistics used did not contain any individual-level information.

### MR assumptions

To be valid, instrumental variables (IVs) used in MR analyses must meet three assumptions (MR IV 1–3): (1) the IVs must be strongly associated with the exposure, (2) the IVs must be independent of all (observed or unobserved) confounders of the exposure-outcome relationship, and (3) the IVs must be associated with the outcome only via the exposure (i.e., independent of the outcome given the exposure; Figure 1A) [38]. The expanded assumptions (multivariable MR IV 1–3) for valid IVs in multivariable MR analyses are that: (1) the IVs must be strongly associated with each exposure given all other exposures in the model, (2) the IVs must be independent of all confounders of any of the relationships between the exposures and the outcome, and (3) the IVs must be independent of the outcome given all exposures (Figure 1B) [38,39].

### Statistical analyses

We used the TwoSampleMR R package v0.6.11 [34], custom functions in R v4.3.2 [40], and PLINK 2.0 alpha v6.5 [41,42] to extract and process exposure GWAS summary statistics, and harmonize the exposure and outcome GWAS summary statistics to create MR instruments. For each exposure GWAS, only SNPs with *p* < 5×10^−8^ were extracted, then SNPs with linkage disequilibrium (LD) were excluded (r^2^ = 0.001 and clumping window = 10,000 base-pairs) using the 1000 Genomes Project European genetic ancestry (1000G EUR) data as an LD reference panel [34,43]. For the multivariable MR instrument, if a SNP was not included in both the CIM and BMI GWAS summary statistics, then a proxy SNP with r^2^ > 0.8 in the 1000G EUR LD reference panel was used [35]. Ambiguous strand information was resolved using allele frequency information when possible. Palindromic SNPs were retained if strand information could be inferred.

When constructing all instruments, we excluded SNPs with a minor allele frequency (MAF) below 5% in the 1000G EUR reference panel [43]. We constructed two instruments for the CRP univariable MR analyses to try to disentangle the influences of CRP from BMI on MD phenotypes. The first instrument (CRP) included SNPs that met the standard inclusion criteria described above. The second instrument (CRP_noBMI) was further restricted to CRP-associated SNPs that did not show an association (*p* ≥ 0.05) with BMI and were not in LD with a BMI-associated SNP (r^2^ > 0.1 within 500 kb) in the 1000G EUR LD reference panel [36,43,44]. The number of SNPs in each of the final instruments are shown in Table S3.

For all CIM instruments, we conducted univariable MR and multivariable MR analyses using random-effect inverse-variance weighted (IVW) methods [45]. These methods were implemented in the MendelianRandomization R package v0.10.0 [46]. Primary inference was based on IVW MR analyses, with nominal p-values providing suggestive evidence in the context of multiple testing across CIM-MD phenotype pairs. A total of 61 CIM instruments with at least one valid SNP were tested using the univariable MR method for each of the three MD outcomes. CRP had two instruments for univariable MR analyses as described above. This resulted in 183 (61 × 3) total univariable MR analyses representing 180 (60 × 3) unique CIM-MD phenotype relationships. For multivariable MR, a total of 60 instruments resulted in 180 total multivariable MR analyses for 180 unique CIM-MD associations. As a result, the corresponding Bonferroni-corrected significance thresholds were *p* < 2.73 × 10^−4^ (0.05 / 183) and *p* < 2.78 × 10^−4^ (0.05 / 180), respectively, for univariable and multivariable IVW MR analyses.

### Assessment of MR assumptions and sensitivity analyses

In univariable MR analyses, we calculated *F*-statistics for each of the instruments to assess the MR IV1 assumption [46]. Although there are no formal tests for the MR IV2 and IV3 assumptions, we used up to seven methods with varying degrees of robustness to help identify potential violations. For instruments with at least one SNP, we calculated MR Wald ratios for each SNP in the instrument. For instruments with more than two SNPs, we further used the MR-Egger [47], MR weighted median (MR median), MR-LASSO [48], and MR constrained maximum-likelihood (MR cML) [49] methods implemented in the MendelianRandomization R package [46]. For instruments with more than 10 SNPs, we used the MR pleiotropy residual sum and outlier (MR-PRESSO) method [50]. The MR-LASSO, MR cML, and MR-PRESSO methods support the identification of outliers and potentially invalid SNPs in the instruments.

To assess the multivariable MR IV1 assumption, we calculated Sanderson Windmeijer conditional *F*-statistics for each exposure in the multivariable MR analyses. For the CRP multivariable MR analyses, we also incorporated the estimated phenotypic correlation between CRP and BMI in UK Biobank (UKB) participants into the conditional *F*-statistic calculations. This phenotypic correlation was estimated by Pearson correlation between CRP ln(mg/l) and BMI (kg/m^2^) at baseline for 466,356 participants with available data. To identify violations of multivariable MR IV2-IV3 and outliers or invalid SNPs, we used multivariable extensions of the MR-Egger, MR weighted median, MR-LASSO, MR-cML, and MR generalized method of moments (MR GMM) methods implemented in the MendelianRandomization R package [34]. For MR cML and multivariable MR cML, the range of potentially invalid IVs was based on the number of potentially invalid IVs detected by MR-LASSO or multivariable MR-LASSO, respectively. Given the strong correlation between BMI and CRP, we used two additional multivariable MR methods for the CRP and BMI (CRP_BMI) analyses: the multivariable MR-PRESSO method implemented in the MR-PRESSO R package [50] and robust Q-statistic minimization method implemented in the multivariable MR (MVMR) R package v0.4.1 [39].

## Results

### Study population

The SNP-exposure effects estimated in the CIM GWAS populations are expected to be similar to those in the MD GWAS population, as all individuals were of inferred European genetic ancestry. None of the BCAC studies included in the MD GWASs were included in any of the CIM GWASs, and any sample overlap is deemed minimal (Tables S1 & S2). The estimated Pearson correlation between CRP ln(mg/l) and BMI (kg/m^2^) at baseline in UKB participants (*N* = 466,356) was r = 0.43.

### Univariable MR analysis

All instruments used in univariable MR analyses had *F*-statistics > 10, suggesting strong instruments (Table S3). All the SNPs included in the CIM instruments were either associated with that CIM or were in high LD (r^2^ > 0.8) with a SNP associated with that CIM in the GWAS Catalog or a previous publication [36,44].

In univariable random-effects IVW MR analyses, we observed nominally statistically significant (*p* < 0.05) associations for CCL23, GDNF, MMP-10, and TRANCE with DA, CD40 and TWEAK with NDA, and GDNF and MMP-10 with PMD (Table 1, Figure 2, & Table S4). For DA, the effect estimates for CCL23 and TRANCE were directionally consistent across multiple MR sensitivity analyses (Figure 2, Tables S4-S9). Similarly, the effect estimate for TWEAK with NDA was generally consistent across sensitivity analyses(Figure 2, Tables S4 & S5). The effect estimates for GDNF and MMP-10 were directionally consistent across DA and PMD analyses (Table S4). The instruments for GDNF, MMP-10, and CD40 each included a single SNP, precluding additional multi-SNP sensitivity analyses.

Across remaining CIMs, univariable IVW effect estimates were generally consistent with those from MR sensitivity analyses (Tables S4-S9). Exceptions were CCL28-NDA, CRP-PMD, and FGF19-PMD, which had significant MR-Egger intercepts, indicating possible horizontal pleiotropy (Table S6). The CRP instrument also had significant MR-PRESSO global test p-values in the NDA and PMD analyses, further indicating possible horizontal pleiotropy for those associations (Table S10). We observed limited evidence of horizontal pleiotropy across most univariable MR analyses. No other CIM instruments were nominally associated with any MD outcome in IVW analyses (all *p* ≥ 0.05). MR-LASSO generally flagged few (0 to 24) SNPs as potentially invalid across instruments (Table S8). MR-PRESSO identified three potential outlier SNPs (rs28429148 and rs4817984) for the CRP instrument in the NDA analysis, two potential outlier SNPs (rs2393794 and rs4817984) for the CRP_noBMI instrument in the NDA analysis, and one outlier SNP (rs28429148) for the CRP instrument in the PMD analysis (Table S10).

### Multivariable MR analysis

In multivariable MR analyses taking BMI into account, conditional *F*-statistics for all exposures except BMI and CRP were *F* < 10, indicating the potential for weak instrument bias (Table S3). In agreement with our previous work [37], we observed strong inverse associations between BMI and PMD (−0.81 sqrt(%) per 1 kg/m^2^ increase in BMI; 95% CI: −1.02, −0.61; *p* = 1.6 × 10^−15^) and DA (−0.35 sqrt(cm^2^) per 1 kg/m^2^ increase in BMI; 95% CI: −0.60, −0.10; *p* = 0.006), and a positive association between increased BMI and NDA (1.34 sqrt(cm^2^) per 1 kg/m^2^ increase in BMI; 95% CI: 0.95, 1.73; *p* = 1.1 × 10^−11^) in the CRP_BMI multivariable MR analysis. The estimated associations between BMI and MD outcomes were generally consistent across multivariable IVW MR models and similar to those observed in the CRP-BMI multivariable MR analysis (Table S4). We observed nominally significant association between CRP and PMD (0.131 sqrt(%) per 1 ln(mg/l) increase in CRP; 95% CI: 0.00, 0.26; *p* = 0.05), but no evidence of association for DA (*p* = 0.24) or NDA (*p* = 0.06) (Figure 3 & Table S4). We also observed nominally statistically significant associations for CCL23 with DA, TRAIL with NDA, and CCL20, OPG, and TRANCE with PMD, with no evidence of horizontal pleiotropy based on multivariable MR-Egger intercepts (Table S6).

## Discussion

The purpose of this study was to investigate the relationships between genetically predicted CIMs and MD phenotypes using an MR approach. In this comprehensive MR analysis of 60 CIMs, we found limited evidence supporting broad causal effects of circulating inflammatory markers on mammographic density phenotypes. Although several nominal associations were observed, none survived correction for multiple testing. Overall, the consistency of largely null findings across the CIMs evaluated suggests that circulating inflammatory markers are unlikely to have broad or strong causal effects on MD phenotypes. The nominal associations identified from multi-SNP instruments in univariable MR analyses (CCL23, TRANCE, and TWEAK) were highly consistent across all of the MR methods we utilized. With the exception of CRP, none of these CIMs have previously been reported to be associated with MD phenotypes, but all have previously been linked to breast cancer biology, including pathways involving immune activation, apoptosis, and tumor progression [14–17,51–59]. However, because none of these observed associations were significant after multiple testing correction, these findings should be viewed as hypothesis-generating and require replication in independent datasets. Notably, CRP was the only CIM with previously reported observational associations with MD phenotypes for which we observed nominal associations in the present study. We did not identify associations (all *p* ≥ 0.05) between genetically predicted concentrations of IL-6, IL-8, TNFR2, or VEGF and any MD phenotype, suggesting that some previously reported observational associations may reflect confounding, reverse causation, or differences between circulating and local tissue-specific inflammatory processes [22–24].

The association between CCL23 and DA was the only association observed in both the univariable and multivariable MR analyses. However, the direction of the observed CCL23-DA association changed from positive in univariable MR to negative in multivariable MR analyses, further reducing confidence that this association reflects a robust causal effect. In addition, the observed negative association for PMD and TRANCE in the multivariable MR analysis conflicts with the observed positive association for DA and TRANCE in the univariable MR analysis (Figures 2 & 3). For all CIM instruments with the exception of CRP, conditional *F* statistics in the multivariable MR analyses indicated weak instruments. In contrast to univariable MR where weak instruments tend to bias estimates towards the null, the direction of bias from weak instruments in multivariable MR is more challenging to predict (39)39. Thus, these multivariable MR analyses should be interpreted cautiously and considered exploratory.

Our results for CRP were inconsistent across MR methods. In univariable IVW analyses, we observed no associations between CRP and MD phenotypes, whereas in multivariable MR IVW analyses including BMI, we observed a nominal positive association between CRP concentrations and PMD. The magnitude of the CRP-PMD association was generally consistent across the multivariable MR analyses. Overall, we found that inclusion of BMI in multivariable MR analyses reduced conditional instrument strength for most CIMs, highlighting the difficulty of disentangling adiposity-related pathways from systemic inflammatory processes and/or theoretical challenges with multivariable MR when the number of genome-wide significant SNPs varies widely between exposures of interest. Given the strong association between BMI and both CRP and MD phenotypes [4,26–28], it is possible that our multivariable MR results better reflect the underlying relationship between CRP and MD phenotypes, especially as the GWAS summary statistics used to construct CRP instruments were not adjusted for BMI. Indeed, it has been proposed that multivariable MR approaches may produce less biased estimates in the absence of confounder-adjusted exposure or outcome GWAS summary statistics, regardless of the bias correction approaches used for univariable MR [60]. Notably, the CRP-PMD association reached nominal significance only in the MVMR analysis, whereas the standard CRP instrument was null and the CRP_noBMI instrument produced a positive but non-significant estimate, suggesting that the estimated direct effect of CRP may be sensitive to how BMI-related genetic variation is accounted for. Further, it is also important to note that previous bidirectional MR studies only estimated the linear effects between BMI and CRP [27,28]. Non-linear relationships between BMI and CRP are possible [17], and could make modeling the BMI-CRP relationship in multivariable MR analyses more complex.

While tumor-promoting inflammation is a cancer hallmark [7,8], there is some evidence to suggest that CIMs may not be good proxies for tissue-specific inflammatory markers. An MR study of the relationships between 66 CIMs and 30 adult cancers (including breast cancer) in a European genetic ancestry population found limited evidence that CIM concentrations were associated with cancer [61]. While both local and systemic immune responses are relevant to breast cancer risk [62], associations between inflammatory marker concentrations and MD measures have generally been stronger in studies that measured levels in breast tissue [22,23] than in studies that measured CIM levels [21,24,25], suggesting that local inflammation may be more directly relevant to MD. Assessing the relationships between tissue-specific inflammatory markers and MD would therefore be informative given the potential relevance of the breast microenvironment to MD and breast cancer [7,8,22,23]. However, we are not aware of studies that provide genetic instruments for breast-specific inflammation markers and were therefore unable to assess these associations here.

Our study has several strengths. To our knowledge, this is the largest and most comprehensive MR analysis of the relationships between CIMs and MD phenotypes conducted to date. We applied multiple MR methods with varying degrees of robustness to violations of MR assumptions and used both univariable and multivariable MR approaches to help mitigate the impact of BMI on CRP estimates.

Our study also has some limitations. First, all GWAS were conducted using European genetic ancestry individuals, limiting the generalizability of results to other populations. Given the global importance of breast cancer, examining these relationships in more genetically diverse populations would be informative. In addition, MR results only represent causal effect estimates when all assumptions are satisfied [38,39,47]. The presence of pleiotropy or invalid SNPs may violate the assumptions required for causal inference. In the case of our multivariable analyses, we observed the potential for weak instrument bias in the instruments for all CIMs except CRP. While we only observed direct evidence of pleiotropy in our sensitivity analyses for a small number of CIMs, we observed evidence of heterogeneity in a larger number of MR analyses. In addition, several of the identified associations were based on single-SNP instruments, limiting our ability to assess potential horizontal pleiotropy through standard MR sensitivity analyses. Finally, the GWAS for all CIMs except CRP had modest sample sizes (N < 55,000), and larger GWAS may provide more informative IVs.

Overall, this study provides limited evidence supporting broad causal effects of circulating inflammatory markers on MD phenotypes. These findings suggest that previously reported observational associations between CIMs and MD may not reflect true causal effects, with the possible exception of CRP, for which we observed a small positive association with PMD in multivariable MR analyses. A small number of markers may warrant follow-up. Replication in independent datasets and evaluation using larger GWAS will help determine whether any of the nominally significant signals identified here represent robust causal associations.

## Supplementary Material

Supporting Information

Table S1: GWAS summary statistic sources and information.

Table S2: Sample sizes and information about BCAC studies included in outcome MD phenotype meta-analysis GWAS summary statistics.

Table S3: MR and MVMR instrument F-statistics and SNPs.

Table S4: IVW MR and MVMR results.

Table S5: Single SNP MR wald ratio results.

Table S6: MR- and MVMR-Egger results.

Table S7: MR and MVMR median method results.

Table S8: MR- and MVMR-LASSO results.

Table S9: MR- and MVMR-cML results.

Table S10: MR- and MVMR-PRESSO results.

Table S11: MVMR GMM estimator results.

Table S12: MVMR Q-statistic minimization method results.

## Figures and Tables

**Figure 1.**
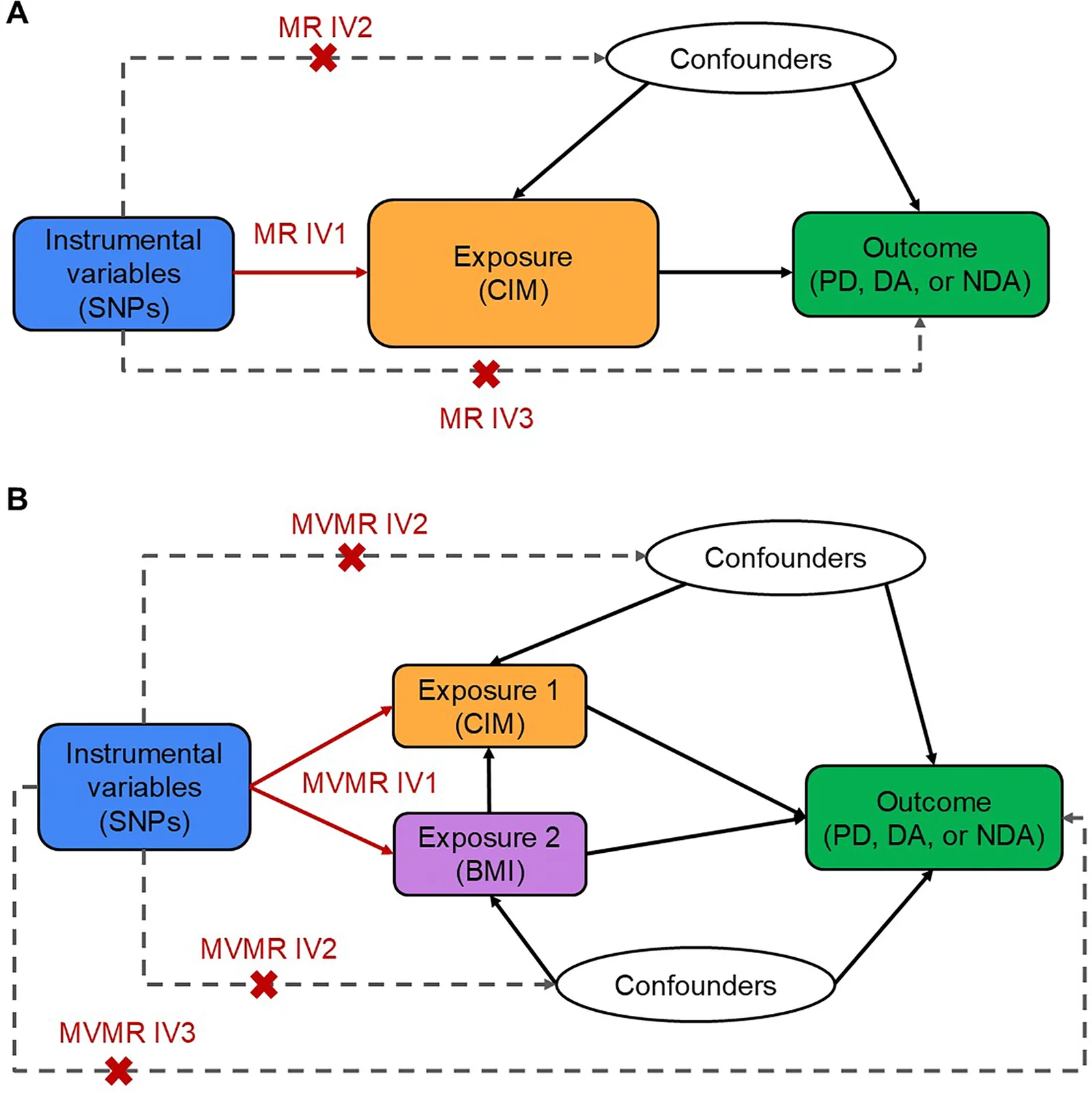
Causal diagrams representing the relationships investigated in Mendelian randomization (MR) and multivariable MR (MVMR) percent mammographic density (PMD), dense mammographic area (DA), and non-dense mammographic area (NDA) analyses with core assumptions indicated. (A) Causal diagram representing univariable MR analyses for CIM exposures and MD outcomes. Core MR instrumental variable (IV) assumptions (MR IV1-IV3) are highlighted in red. (B) Causal diagram representing CRP MVMR analyses for MD outcomes. Core MVMR IV assumptions (MVMR IV1-IV3) are highlighted in red.

**Figure 2.**
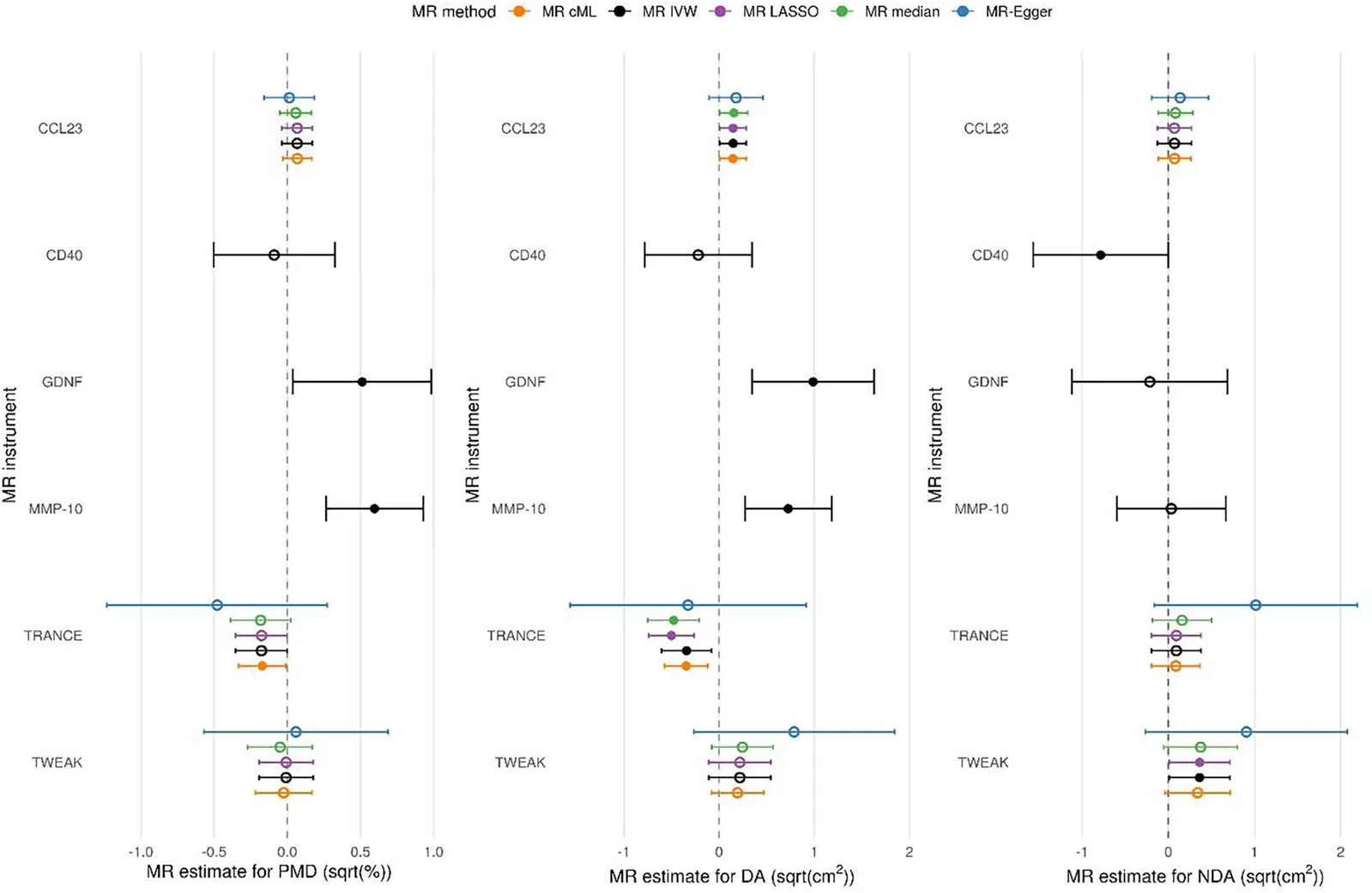
Mendelian randomization (MR) sensitivity analysis results for CIMs with significant MR IVW results for any of the mammographic density phenotypes. CIM instruments with at least one significant (*p* < 0.05) association with an MD phenotype are listed on the y-axes. MR estimates and 95% confidence intervals for PMD, DA, and NDA are on the x-axes. MR methods are indicated by the point and error bar color, with the Egger, median, LASSO, IVW, and cML MR methods plotted from top to bottom for each CIM. Open circles represent MR estimates with *p* ≥ 0.05. Instruments for CD40, GDNF, and MMP-10 only included a single SNP and no additional sensitivity analyses could be performed. Instruments not constructed using summary statistics from the large GWAS of 91 CIMs are indicated with an asterisk.

**Figure 3.**
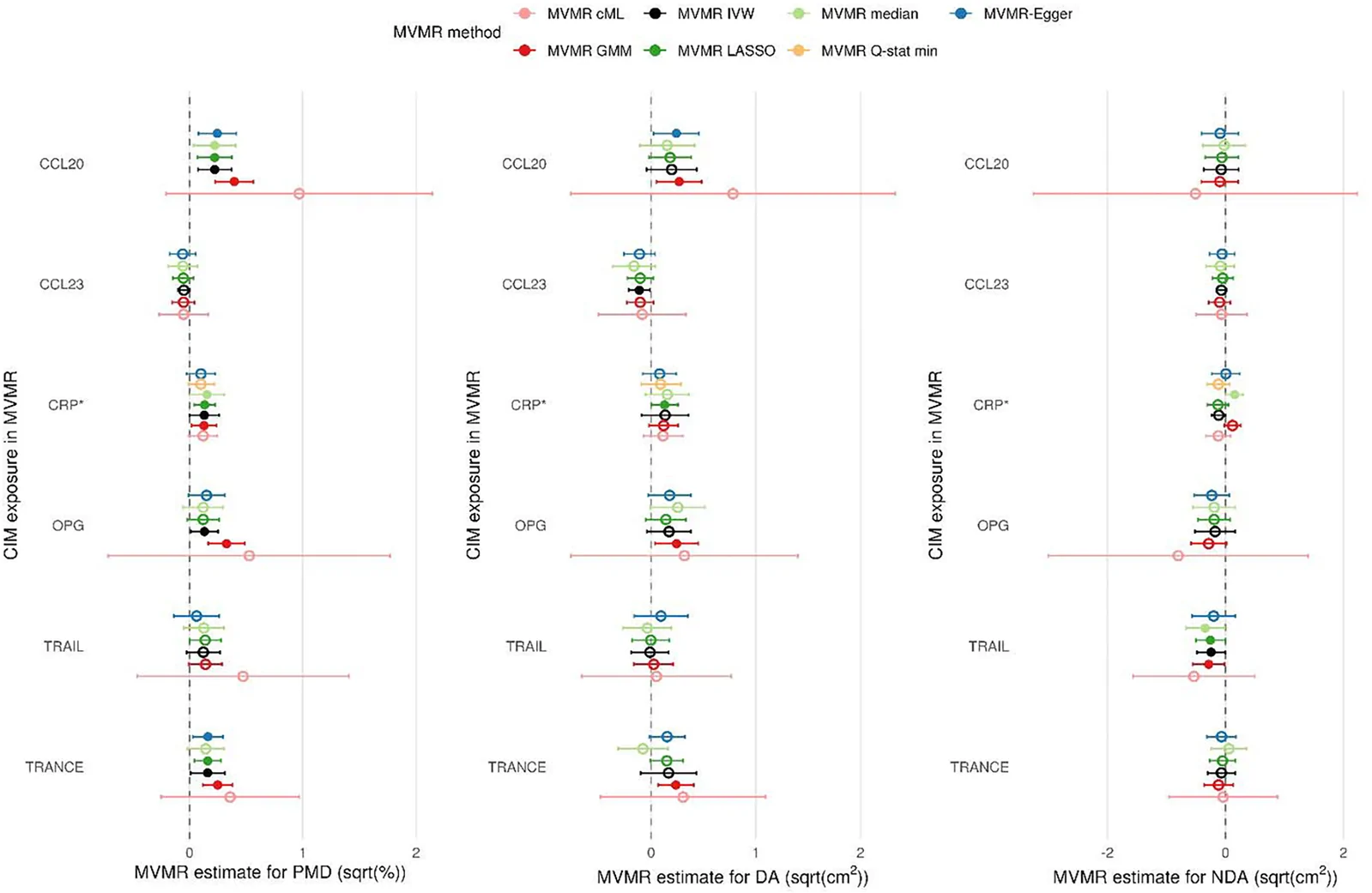
Multivariable Mendelian randomization (MVMR) sensitivity analysis results for CIMs with significant MVMR IVW results for any of the mammographic density phenotypes. CIM exposures in MVMR instruments with at least one significant (*p* < 0.05) association with an MD phenotype are listed on the y-axes. MR estimates and 95% confidence intervals for PMD, DA, and NDA are on the x-axes. MVMR methods are indicated by the point and error bar color, with the Egger, median, LASSO, IVW, and cML MVMR methods plotted from top to bottom for each CIM. Open circles represent MR estimates with *p* ≥ 0.05. The CRP exposure has additional results from the Q-statistic minimization (MVMR Q-stat min) method listed second from the top. Instruments not constructed using summary statistics from the large GWAS of 91 CIMs are indicated with an asterisk.

**Table 1.** Nominal associations between genetically predicted circulating inflammatory markers and mammographic density phenotypes in IVW univariable and multivariable MR analyses.

Exposure^1^	Outcome^2^	Univariable MR				Multivariable MR			

		*N* SNP		95% CI	*p*-value	*N* SNP		95% CI	*p*-value

CCL23	DA	3	0.15	0.01, 0.28	0.039	275	−0.11	−0.21, −0.01	0.032
GDNF	DA	1	0.99	0.35, 1.63	0.003	272	0.10	−0.12, 0.32	0.387
MMP-10	DA	1	0.73	0.27, 1.18	0.002	272	−0.04	−0.29, 0.21	0.769
TRANCE	DA	5	−0.34	−0.60, −0.08	0.010	277	0.17	−0.10, 0.43	0.216
CD40	NDA	1	−0.78	1.57, −0.0005	0.049	272	−0.10	−0.51, 0.31	0.631
TWEAK	NDA	4	0.36	0.01, 0.71	0.042	273	−0.36	−0.83, 0.11	0.132
CRP^3^	PMD	59	0.14	−0.03, 0.31	0.098	281	0.13	0.0003, 0.26	0.049
GDNF	PMD	1	0.51	0.04, 0.99	0.034	272	0.13	−0.04, 0.30	0.137
MMP-10	PMD	1	0.60	0.26, 0.93	<0.001	272	0.05	−0.18, 0.27	0.678

1 Exposure units were ln(mg/l) for CRP and rank-based inverse normalized log2 (pg/ml) for all other CIMs

2 Outcome units were sqrt(cm^2^) for DA and NDA and sqrt(%) for PMD

3 The results shown here are for the CRP_noBMI instrument in univariable MR and CRP_BMI instrument for multivariable MR

## Data Availability

We used publicly available GWAS summary statistics from individual study, GWAS Catalog, and IEU OpenGWAS project databases for circulating inflammatory markers and BMI as cited. The BCAC data used to generate the mammographic density GWAS summary statistics used in this study are available via application to the Data Access and Co-ordination Committee (BCAC@medschl.cam.ac.uk).

## Code Availability Statement

Code used in these analyses are publicly available in our project GitHub repository (https://github.com/UW-Epidemiology/MD_CIM_MR).
